# Enhanced Lacto-Tri-Peptide Bio-Availability by Co-Ingestion of Macronutrients

**DOI:** 10.1371/journal.pone.0130638

**Published:** 2015-06-22

**Authors:** Gabriella A. M. Ten Have, Pieter C. van der Pijl, Arie K. Kies, Nicolaas E. P. Deutz

**Affiliations:** 1 Center for Translational Research in Aging & Longevity, Department of Health & Kinesiology, Texas A&M University, College Station, TX, United States of America; 2 Department of Surgery, Nutrition and Toxicology Institute Maastricht, Maastricht University, Maastricht, The Netherlands; 3 Unilever Research & Development, Vlaardingen, The Netherlands; 4 DSM Biotechnology Center, Delft, The Netherlands; Radboud university medical center, NETHERLANDS

## Abstract

Some food-derived peptides possess bioactive properties, and may affect health positively. For example, the C-terminal lacto-tri-peptides Ile-Pro-Pro (IPP), Leu-Pro-Pro (LPP) and Val-Pro-Pro (VPP) (together named here XPP) are described to lower blood pressure. The bioactivity depends on their availability at the site of action. Quantitative trans-organ availability/kinetic measurements will provide more insight in C-terminal tri-peptides behavior in the body. We hypothesize that the composition of the meal will modify their systemic availability. We studied trans-organ XPP fluxes in catheterized pigs (25 kg; n=10) to determine systemic and portal availability, as well as renal and hepatic uptake of a water-based single dose of synthetic XPP and a XPP containing protein matrix (casein hydrolyte, CasH). In a second experiment (n=10), we compared the CasH-containing protein matrix with a CasH-containing meal matrix and the modifying effects of macronutrients in a meal on the availability (high carbohydrates, low quality protein, high fat, and fiber). Portal availability of synthetic XPP was 0.08 ± 0.01% of intake and increased when a protein matrix was present (respectively 3.1, 1.8 and 83 times for IPP, LPP and VPP). Difference between individual XPP was probably due to release from longer peptides. CasH prolonged portal bioavailability with 18 min (absorption half-life, synthetic XPP: 15 ± 2 min, CasH: 33 ± 3 min, p<0.0001) and increased systemic elimination with 20 min (synthetic XPP: 12 ± 2 min; CasH: 32 ± 3 min, p<0.0001). Subsequent renal and hepatic uptake is about 75% of the portal release. A meal containing CasH, increased portal 1.8 and systemic bioavailability 1.2 times. Low protein quality and fiber increased XPP systemic bioavailability further (respectively 1.5 and 1.4 times). We conclude that the amount and quality of the protein, and the presence of fiber in a meal, are the main factors that increase the systemic bioavailability of food-derived XPP.

## Introduction

In the recent years our understanding of potential food-derived bioactive peptides and their potential health benefits advanced [1,2], mainly on the role of bioactive peptides from milk. For instance, the effects on blood pressure of the lacto-tri-peptides Isoleucine-Proline-Proline (IPP) and Valine-Proline-Proline (VPP) is described in animal [3] and human studies [4–12]. The hypothesized working mechanism of these peptides is inhibition of the angiotensin I converting (ACE) enzyme [4] and is related to the presence of a proline residue on the C-terminal of the lacto-tri-peptides [13]. Animal and human studies also suggest that IPP and VPP reduce arterial stiffness and improve endothelial activity [14,15].

The beneficial effects of food-derived bioactive peptides depend on availability on the site of action. For instance, to exert a potential ACE inhibition effect after oral intake of the lacto-tri-peptides, the peptides have to reach the cardiovascular system in an active form [16]. Metabolic active organs like the intestine, liver, and kidney play a major role in the availability. Fluxes of these peptides through those organs are likely to be dependent on the fluxes of other macronutrients, especially proteins and amino acids [17–22]. No fundamental knowledge of quantitative organ flux data are available for food-derived bioactive lacto-tri-peptides to get more insight in the behavior of those peptides in the body [23]. Therefore, the main aim of the present study is to collect quantitative data about how much food-derived bioactive lacto-tri-peptides is absorbed and released to the portal system, the role of kidney and liver and their effect on the systemic availability under different nutritional conditions. We studied IPP, VPP and LPP (Leucine-Proline-Proline), named together as XPP as model for C-terminal proline containing food-derived peptides in general, because they are relatively resistant against breakdown in the gastro-intestinal tract and therefore expected to be similar in their kinetics [3,24].

Previously, we found that the systemic bioavailability of a single dose of synthetic XPP, when given orally as a single dose, was less than 0.1% [25] with a half-life maximum at 15 min. In the present study, we hypothesize that dietary composition modifications improve absorption kinetics of XPPs and thus their bioavailability.

Therefore, we studied trans-organ XPP fluxes in catheterized pigs to determine the effect of a protein matrix on the XPP systemic and portal availability, renal and hepatic uptake using a XPP containing casein hydrolyte (CasH). Casein is the main lacto-protein that is rich of encrypted XPP’s (www.genome.jp). This, in contrast with the lacto-protein whey or a non-milk protein soy with no encrypted XPP’s. The studied CasH is rich of liberated IPP and LPP, produced by using enzymes including a specific prolyl-endoprotease [26]. This CasH is proven to have ACE inhibitory potential [12] and has a faster digestion and absorption rate [27] than casein, which potentially relates to the maximally available systemic XPP concentrations. In a second experiment, we studied the potential effect on XPP availability when the XPP containing CasH was added to a meal and studied the potential modifying effects of macro-nutrients (high carbohydrates, low quality protein, high fat, and fiber) in the meal. Quantification of intestinal absorption kinetics of XPP and the role of liver and kidney is not possible in humans, therefore we measured the transorgan XPP fluxes in conscious multi-catheterized pigs [28,29] as the gastro-intestinal tract of the pig is comparable with that of humans [30–32].

The quantitative results of the present study are important as crucial fundamental information for the development of therapies with bioactive peptides.

## Material and Methods

### Animals

For each study, we used 10 pathogen-free, female piglets (Dutch landrace x Yorkshire; 8–12 weeks of age; 25.2 ± 1.1 kg body weight) that were adapted to individual housing in straw containing pens (2 x 3 m) with 12h day-night cycle and an environmental temperature of 23°C one week before surgery. Catheters for blood sampling were placed during surgery [28] under isoflurane (2% mixture with oxygen) anesthesia and flunixin meglumine (50 mg/25 kg bw) analgesia, in the abdominal aorta, the portal vein, the hepatic vein, and the renal vein. Catheters for infusions of post-operative medication or *para*-aminohippuric acid solution (PAH; flow measurements) were placed in the central vein and splenic vein and for gastric administration a gastric catheter. We checked the position of the catheter tips with X-ray during surgery using fluoroscopy and an iodine containing contrast solution.

During the recovery period (7–10 days), twice daily, we and the assigned veterinarian of the animal care facility monitored the animals for general behavior, body temperature, food intake, body weight and catheter patency and administered buprenorphine (0.03mg/kg) analgesia, *intra-venous*, twice daily on day 1–4 and when needed. Animals were accustomed to a small movable cage (0.9 x 0.5 x 0.3 m) to perform the experiments. At all times, animals would be euthanized with 195 mg/kg bw pentobarbital sodium and 25 mg/kg bw phenytoin sulfate, *intra-venous*, if imminent death is expected.

#### Ethics Statement

The animal ethics committee of Maastricht University, The Netherlands approved the studies: 2004–101. We performed the surgery under isoflurane anesthesia, and all efforts were made to minimize suffering. The researchers and the assigned veterinarian of the animal care facility monitored the anesthesia and recovery closely.

### Experimental protocol

#### Study 1

After a postoperative recovery period of 7–10 days, we conducted five test days with XPP mixtures according to a Williams cross-over design [33] with a minimum of 2 days (washout) between the test days. One *intra-venous* administered IPP, LPP and VPP mixture (data previously published [25]) and four *intra-gastric* administered XPP mixtures were studied (Table 1). We studied a control solution (Control-group) without XPP, but iso-ionogenic to the other mixtures for sodium, potassium and calcium ions, to determine potential occurring endogenous XPP fluxes. We gave a water-based matrix with comparable amounts of IPP, LPP and VPP (XPP-group) for comparison with a protein based XPP mixture (casein hydrolyte, CasH-group). The CasH contained known amounts of free XPP with a C-terminal proline. We gave a XPP spiked protein matrix (CasH+XPP group) to evaluate the influence of a protein matrix on individual XPP’s. In the present study, we only will discuss the data of the four *intra-gastric* administered XPP. We administered these mixtures as a single bolus with an end volume of 14 ml/kg body weight and temperature of 37°C.

**Table 1:** Composition of test mixtures—Study 1.

*Substance*	*Unit*	*Control*	*XPP* ^*1*^	*CasH* ^*2*^	*CasH* ^*2*^ *+XPP* ^*1*^
**IPP**	μmol/kg bw	0.0	12.3	10.7	23.0
**LPP**	μmol/kg bw	0.0	12.3	32.9	45.2
**VPP**	μmol/kg bw	0.0	12.8	0.62	13.4
**Total XPP** ^**3**^	μmol/kg bw	0.0	37.4	44.2	81.6
**Protein**	mg/kg bw	0.0	0.0	648	648
**Sodium**	mM	3.01	3.01	3.01	3.01
**Potassium**	mM	71.14	71.14	71.14	71.14
**Calcium**	mM	1.96	1.96	1.96	1.96
**Phosphate**	mM	71.1	71.1	102.9	102.9

^1)^ Chemical purities of the IPP, LPP, and VPP synthetic products were 93.4, 95.0 and 98.7%, respectively (Bachem, Weil am Rhein, Switzerland).

^2)^ The given amounts of tri-peptides isoleucine-proline-proline (IPP), leucine-proline-proline (LPP), valine-proline-proline (VPP) in the casein hydrolysate (CasH, Casimax, DSM Food Specialties, Delft, The Netherlands). The casein hydrolysate contained 57% protein with 5.4, 16.5 and 0.3 mg/g protein of LPP, LPP and VPP, respectively.

^3)^ Total XPP = total amount of IPP, LPP and VPP.

#### Study 2

After a postoperative recovery period of 7–10 days, we conducted six test days with CasH interventions according to a Williams cross-over design [33] with a minimum of 2 days (washout) between the test days. We compared a CasH supplemented meal matrix (Basal-group) to a CasH only protein matrix (CasH2-group). We also compared four other CasH supplemented meals with low protein quality (LQprot-group), high carbohydrate (hCHO-group), high fat (hFat-group) and fiber containing Basal meal (Fiber-group) to Basal to evaluate the influence of macronutrients (Table 2). The Basal meal contains a ratio of 25:50:25 En% of the macronutrients protein, carbohydrates and fat. All meals were iso-caloric (30% of daily intake: 37.2 kcal/kg bodyweight) and composed by Research Diet Services (Wijk bij Duurstede, The Netherlands). The protein fraction in the meals was a high quality whey protein isolate, while in the low quality protein diet a soy protein isolate that had 24% less essential amino acids. We gave all meals *intra-gastric* as a single bolus with an end volume of 24 ml/kg bodyweight and 37°C and observed for 360 min.

**Table 2:** Composition of test meals—Study 2.

	*Nutrient*	*Units*	*CasH2*	*Basal*	*LQprot*	*hCHO*	*hFat*	*Fiber*
**Meal**	Whey protein	Energy %	0	25	0	25	25	25
	Soy protein	Energy %	0	0	25	0	0	0
	CHO	Energy %	0	50	50	65	25	50
	Fat	Energy %	0	25	25	10	50	25
	Total energy intake	kcal/kg bw	0	37.2	37.2	37.2	37.2	37.2
	Whey protein ^1^	g/kg bw	0	2.32	0	2.32	2.32	2.32
	Soy protein ^1^	g/kg bw	0	0	2.32	0	0	0
	CHO ^2^	g/kg bw	0	4.64	4.64	6.04	2.32	4.64
	Fat ^3^	g/kg bw	0	1.04	1.04	0.42	2.07	1.04
**Supplement**	Casein hydrolysate ^4^	g/kg bw	0.73	0.73	0.73	0.73	0.73	0.73
	Fiber ^5^	g/kg bw	0	0	0	0	0	0.18
**Gross weight**		g/kg bw	1.28	9.80	9.94	10.7	8.32	9.99
**End Volume**		ml/kg bw	24	24	24	24	24	24

^1)^: Indicated is the amount of protein supplied by the isolates.

^2)^: CHO: maize starch, sucrose and glucose in the weight ratio 2:1:1.

^3)^: Fat: soybean oil and sunflower oil in the weight ratio 4:1.

^4)^ The casein hydrolysate product (Casimax, DSM Food Specialties, Delft, The Netherlands) contained 57% protein with 5.4, 16.5 and 0.3 mg/g protein LPP, LPP and VPP, respectively.

^5)^ Fiber: modified citrus pectin.

Sixteen hours before each test day, we removed food. Plasma flow determination started 1h before feeding (t = 0) and continued throughout the experiment by a primed-continuous infusion of *para*-aminohippuric acid through the splenic infusion catheter. At steady state concentrations of PAH (1 hour [28]), we took blood samples from the arterial, portal, hepatic, and renal vein. We gave the liquid test mixture or meal *via* the gastric catheter within 5 min. Subsequently, we took blood samples (1.5 ml/sample) in study 1 from the arterial, portal, hepatic and renal catheter at T = 1, 3, 5, 7, 9, 12.5, 17.5, 25, 30, 40, 50, 60 and 90 min; in study 2 from the arterial and portal catheter at T = 10, 20, 40, 60, 90, 120, 150, 180, 210, 240, 270, 300, 330 and 360 min.

### Sample processing

Directly after collection in Lithium-Heparin tubes, we placed the blood samples on ice. We processed all blood samples within 1 hour after collection to ensure stability of the substances. We centrifuged at 4°C for 15 min at 3000 g to obtain plasma. For PAH concentration determinations we added 40 μl of 50% w/v *Tri*-chloroacetic acid solution (TCA) to 400 μl of plasma and mixed. For XPP analyses, we added 10 μl of 10% w/v *tri-*fluoroacetic acid (TFA) to 500 μl plasma and mixed. We froze the samples in liquid nitrogen and stored at -80°C until further analysis. We took samples from the PAH infusion solutions and the test mixtures.

### Biochemical analyses

We analyzed the plasma samples and test mixtures for the peptides IPP, LPP and VPP (XPP) content [25,34]. We added 50 μl internal standard solution, containing U-^13^C-IPP, U-^13^C-VPP, and U-^13^C-LPP to homogenized plasma (20 μl) and 480 μl water. After mixing, we acidified this aliquot with *tri-*fluoroacetic acid to pH <3. We removed proteins by heating the aliquot at 95°C for 2 min, followed by centrifugation at 22,000 g for 30 min at 15°C. We quantified the XPP’s in the supernatant with LC-MS (Quattro Ultima, Waters, Milford, MA, USA). The detection limit of quantification of this procedure was 0.28, 0.28, and 0.71 nM for IPP, LPP, and VPP respectively.

We tested samples of test mixtures by mixing 100 μl of the sample with 100 μl of a standard solution of universally ^13^C- labeled IPP [U-13C-IPP] and VPP [U-^13^C-VPP] (Biopeptide Co., San Diego, CA, USA). Then we mixed for 1 min, followed by centrifugation for 20 min at 16,000 x g at room temperature. This procedure allows determination of XPPs in the test mixtures between 1.9 and 123 μM.

We analyzed in a pilot set of plasma samples from Study 1, the occurrence of di-peptides with proline (Proline-Proline (PP), leucine (LP), valine (VP), isoleucine (IP)). We determined di-peptide concentrations with an automated LC-ESI-MS system (QTrap 5500 MS (AB Sciex, Foster City, CA, USA) with ExpressHT Ultra LC (Eksigent Div., AB Sciex, Foster City, CA, USA). We added supernatant (20 μl) of centrifuged TCA deproteinized plasma to a 0.1 N hydrochloric acid containing internal standard (20 μl) of a stable isotopomer [D8-valine, D5-proline]. We derivatized internal standard containing samples and di-peptide external standards with 9-Fluorenylmethoxycarbonyl (Fmoc), neutralized and injected 160 nL of the solution on a micro LC column 0.5 x 100 mm HALO C18, 2.7 um, 90A pores at 35°C. We eluted the analytes with a segmentally linear gradient from 35% to 85% acetonitrile in water supplemented with ammonium acetate to 10 μM and 5% isopropanol. We detected by electrospray triple quadrupole tandem mass spectrometry in multiple reactions monitoring mode. For concentration calculations of di-peptides, we normalized MS signals of the samples and the external standards with their internal standard. We determined di-peptide concentrations with the calibration curve of the external standards.

We determined plasma PAH concentrations with a standard enzymatic method on an automatic spectrophotometric analyzer (Cobas Mira, Hoffmann-La Roche, Basel, Switzerland) as described [22,30–32]. In brief, we measured plasma PAH concentration at 465 nm using Ehrlich’s reagent: 1 g *p-*dimethylaminobenzaldehyde (Merck, Darmstadt, Germany), 35 ml alcohol 96%, 4 ml TCA 2N, 61 ml water (bi-distilled) and a solution of 35% alcohol.

### Calculations

#### Systemic pharmacokinetics

Using a 1-compartment model [25] we determined in study 1, absorption half-life (t_½,a_;min), time to maximum plasma concentration (t_max_;min), maximum plasma concentration (C_max_;nM), elimination half-life (t_½,e_;min) and systemic absolute bioavailability (f_Abs_;%). For study 2, we used a non-compartmental analysis calculate AUCs of the arterial XPP time curves from 0 to 360 min (nmol/l*min). We calculated the systemic bioavailability relative to regular, basal meal using the following equation:

$$f_{\text{Rel}}\;=\;\frac{AUC_{\text{Basal}}\;\cdot \;D_{\text{Test}}}{AUC_{\text{Test}}\;\cdot \;D_{\text{Basal}}}\;\cdot \;100\;\;\%$$

where f_Rel_ = the bioavailability for a administered XPP from a meal relative to Basal (%), AUC_Basal_ = the AUC for Basal (nmol/l*min), D_Test_ = the dose of an XPP in the meal (nmol), AUC_Test_ = the AUC for the meal (nmol/l*min), D_Basal_ = the XPP dose of Basal (nmol).

#### XPP organ fluxes

We measured plasma flow, necessary to calculate amino acid fluxes across organs. We calculated PDV fluxes of IPP, LPP and VPP by portal-arterial XPP concentration difference times portal plasma flow, liver fluxes by subtracting PDV flux from splanchnic flux (hepatic-arterial difference* liver plasma flow) and renal flux by renal plasma flow times renal-arterial XPP concentration difference [22,28,31,32,35]. We expressed organ flux as pmol/kg body weight/min and a positive flux is net release of XPP and negative flux net uptake.

We calculated total net release or uptake (= organ net balance) of XPP across an organ from the area under curve of the respective organ flux time course. We expressed total net balance as pmol/kg bodyweight. For the PDV, the portal bioavailability is the total net balance as fraction dose of IPP, LPP or VPP in the test mixtures/meals. We calculated the free available XPP in the test mixtures of study 1 or test meals in study 2 from information, made available by the manufacturer.

### Statistical procedures

We used Prism 6 (Graphpad Software, La Jolla, CA, USA) for statistics, expressed the results as means ± SEM and set the level of significance on p<0.05 and tendencies are defined as p<0.01. We tested the data for normality and used the Wilcoxon test to determine if means are different from zero.

Comparisons between matrixes and mean XPP responses where done with two-way ANOVA. When appropriate, followed by post-hoc test (Sidak’s, Dunnett’s, Fisher’s LSD or Wilcoxon Signed Rank test as indicated in tables and figures) for planned physiological relevant multiple comparison.

We compared the means of two groups with unpaired t-test or Wilcoxon matched pairs signed rank test, means of more than three groups with One-way repeated measures ANOVA. When appropriate, followed by a post-hoc test (Dunnett’s) for planned physiological relevant multiple comparison.

We compared means of groups in the time courses with two-way ANOVA test, mixed model: repeated measurements for time (fixed effect) but not for test mixtures, for planned physiological relevant comparisons.

## Results

### Effect of a protein matrix on systemic bioavailability and pharmacokinetics of XPP peptides

**Fig 1:**
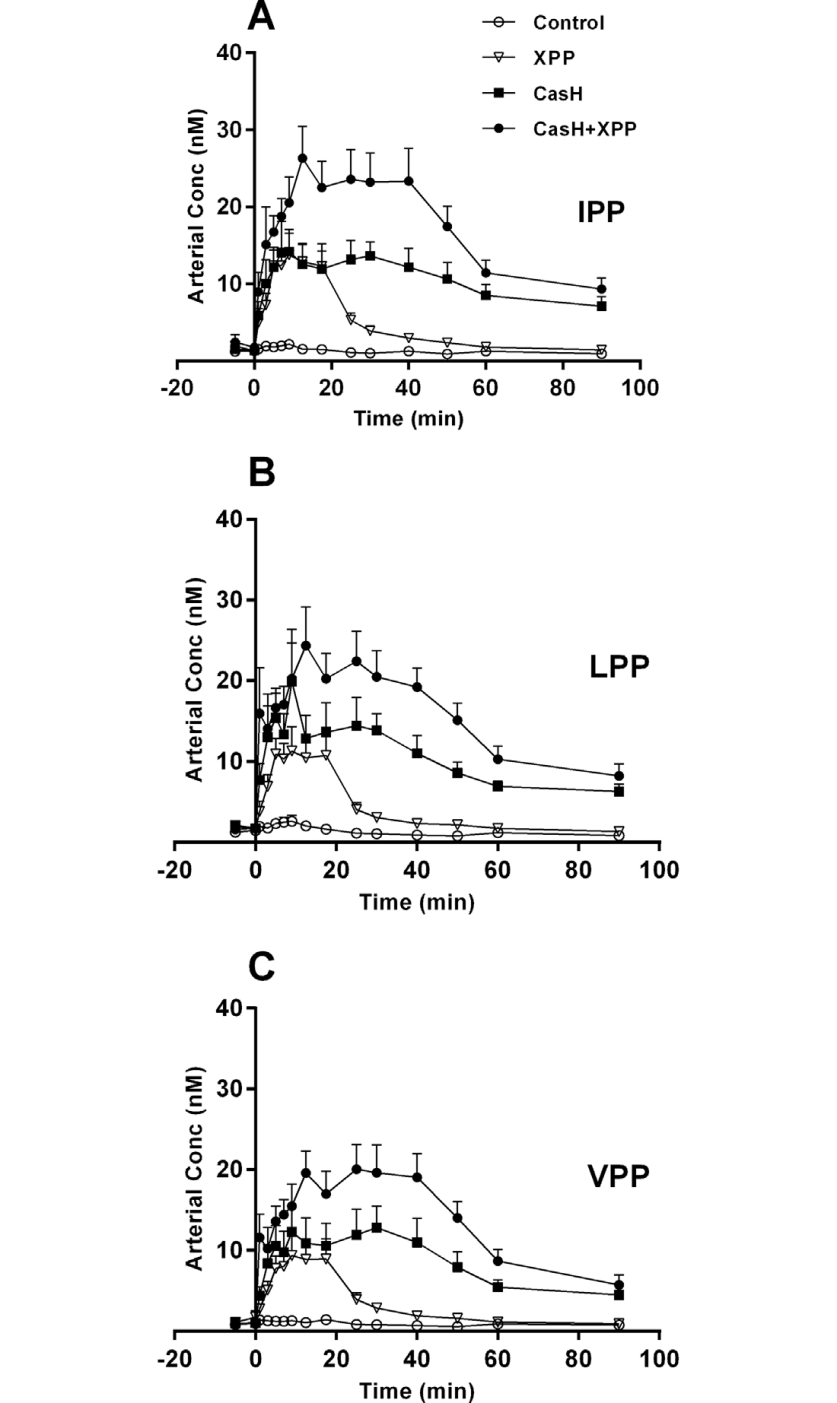
Systemic levels of XPP peptides—Effect of protein matrix. Post-prandial arterial concentrations after intra-gastric administration of control salt solution (Control), synthetic XPP’s (XPP), casein hydrolysate rich in XPP (CasH) and spiked CasH (CasH + XPP). A: Isoleucine-proline-proline (IPP). B: Leucine-proline-proline (LPP). C: Valine-proline-proline (VPP). Respective number of observations for Control, XPP, CasH and CasH +XPP are for graph A: n = 9, 10, 9 and 10; graph B: n = 10, 10, 9 and 10; graph C: n = 8, 10, 9 and 10. Values are mean ± SEM. Statistics: repeated measures two-way ANOVA, mixed model, planned comparisons. All curves are significantly different from the XPP mixture: effect test mixture p<0.001; effect time p<0.001; interaction p<0.001.

Systemic (= arterial) concentrations of XPP in time (Fig 1) showed no measurable baseline/post-absorptive endogenous XPP (Control group, tested with Wilcoxon). The XPP concentrations in the CasH groups were not back to baseline at 90 min post-prandial (one-way ANOVA with Dunnett’s post-hoc test: IPP p<0.002; LPP p<0.001; VPP p<0.05). The calculated systemic absolute bioavailability (f_abs_ (%) = fraction dose absorbed, Table 3) was increased in the protein based CasH group (IPP: 4.7 times, p<0.0004; LPP: 2.5 times, p<0.002; VPP: 121 times, p<0.0001). The spiked CasH group (CasH+XPP) showed lower systemic bioavailability for VPP (65 times, p<0.0001) in comparison to CasH. There was a delay in the time to maximum XPP plasma concentration (t_max_) with the CasH groups compared to the XPP test mixture (12 min, p<0.0001, Wilcoxon matched pairs signed rank test). The calculated absorption half-life (*t*_*½*,*a*_) and elimination half-life (*t*_*½*,*e*_) was delayed significantly in CasH groups (resp. 6 min and 20 min, p<0.0001, Wilcoxon matched pairs signed rank test). Plasma concentrations of the dipeptides LP, IP, VP and PP in the post-prandial period, measured in a limited set of samples, were in the range of 1–7 μM, compared to 1–30 nM for XPP’s in this sample set (S1 Table. Dipeptide/tripeptide ratio—Study 1.).

**Table 3:** Systemic bioavailability and pharmacokinetics of XPP peptides—Effect of a protein matrix.

						*p-value* ^*2*^		
*Parameter*	*Matrix*	*Group*	*IPP*	*LPP*	*VPP*	*Matrix/Spike effect*	*XPP effect*	*Inter-action*
***f***_***abs***_ ***(%)***	*Water-based*	XPP^1^	0.08±0.01	0.06±0.01	0.07±0.02	<0.0001	<0.0001	<0.0001
	*protein*	CasH	0.38±0.05*	0.15±0.03*	8.49±0.69*	<0.0001	<0.0001	<0.0001
		CasH+XPP	0.26±0.06^#^	0.16±0.04	0.13±0.78^#^			
***t***_***½*,*a***_ ***(min)***	*Water-based*	XPP^1^	3.3±0.5	2.0±0.5	4.6±1.1	0.003	0.687	0.581
	*protein*	CasH	11.2±3.3	7.8±2.3	8.5±2.5	0.978	0.789	0.697
		CasH+XPP	7.8±3.6	8.0±2.6	10.5±2.8			
***t***_***max***_ ***(min)***	*Water-based*	XPP^1^	8.6±0.6	7.1±0.7	8.9±0.6	<0.0001	0.628	0.805
	*protein*	CasH	22±5	19±2	19±2	0.775	0.771	0.818
		CasH+XPP	19±5	19±3	20±3			
***C***_***max***_ ***(nM)***	*Water-based*	XPP^1^	12±3	11±3	9±2	0.026	0.823	0.762
	*protein*	CasH	16±3	17±3	16±3	0.022	0.905	0.744
		CasH+XPP	25±3	21±3	21±3			
***t***_***½*,*e***_ ***(min)***	*Water-based*	XPP^1^	9.3±1.1	15±4	12±6	<0.0001	0.590	0.192
	*protein*	CasH	39±5	31±11	27±4	0.970	0.236	0.462
		CasH+XPP	36±6	42±12	23±4			

Pharmacokinetic parameters derived from the systemic response of an *intra gastric* bolus administrated of the tri-peptides isoleucine-proline-proline (IPP), leucine-proline-proline (LPP), valine-proline-proline (VPP) in water-based matrix (synthetic XPP) or in a protein matrix (casein hydrolysate rich in XPP: CasH). f_abs_ (%): systemic absolute bioavailability, t_½,a_: absorption half-life, tmax: time to maximum plasma concentration, Cmax: maximum plasma concentration, t½,e: elimination half-life. Parameters calculated with a 1-compartment model. Values are means ± SEM. For f_abs_ n = 8. For other parameters XPP: n = 10; CasH: n = 10; CasH+XPP: n = 9. Significance: p<0.05.

^1)^: Data XPP group from the present study 1, are published in van der Pijl et al. [25].

^2)^: Significance for comparison of water-based matrix (XPP) with protein matrix (CasH). Or significance for comparison between XPP spiked (CasH+XPP) and non-spiked protein matrix (CasH): Two-way ANOVA. When appropriate, a post-hoc unpaired t-test is done:

*): p<0.05 significance for comparison IPP, LPP or VPP of XPP relative to CasH.

^#)^: p<0.05 significance for comparison IPP, LPP or VPP of CasH relative to CasH+ XPP.

### Effect of meal matrix on portal availability of XPP peptides

**Fig 2:**
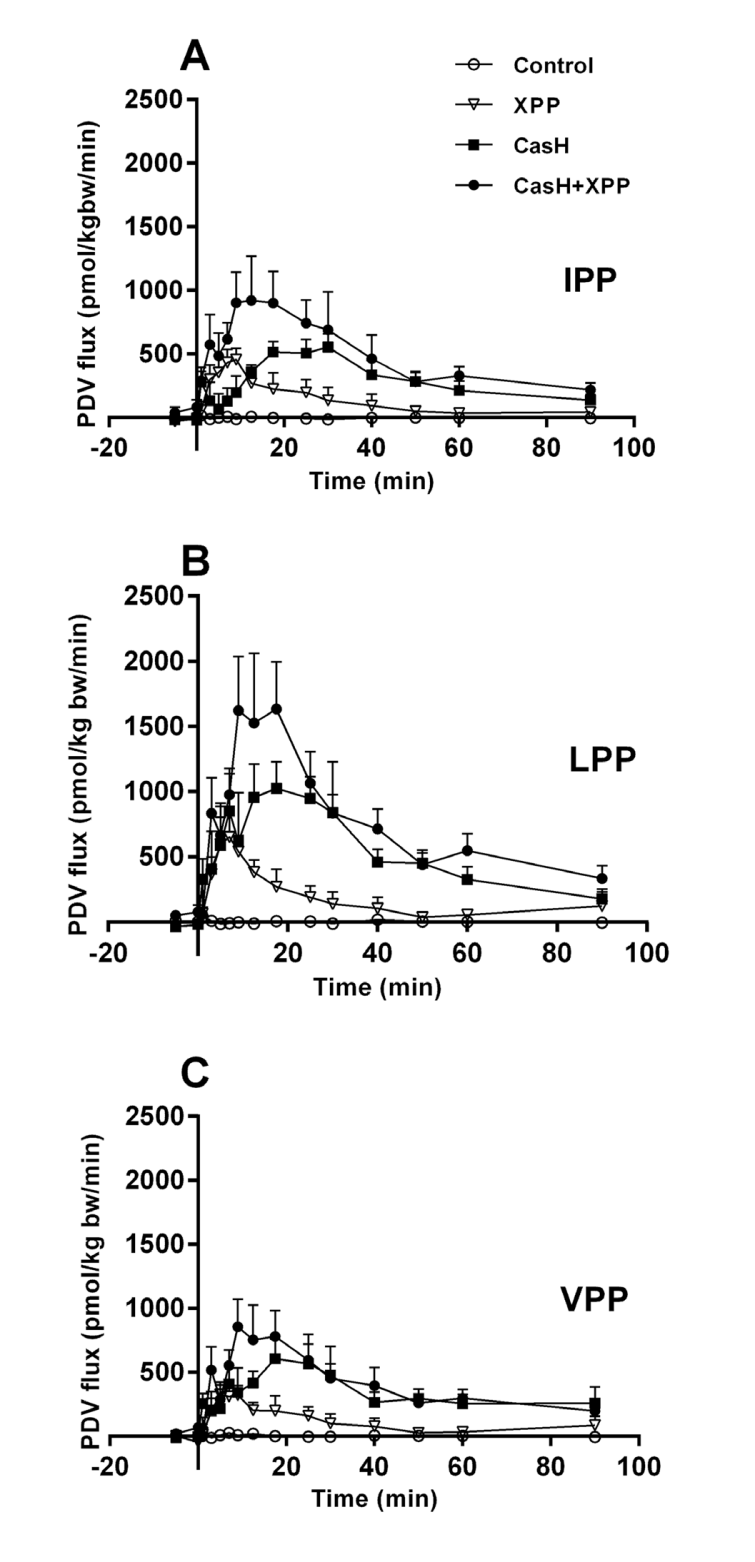
Portal Drained Viscera fluxes of XPP—Effect of a protein matrix. Post-prandial portal drained viscera (PDV) fluxes after *intra-gastric* administration of tri-peptide (XPP) mixtures: control salt solution (Control), synthetic XPP’s (XPP), casein hydrolyte rich in XPP (CasH) or spiked CasH (CasH + XPP). A: Isoleucine-proline-proline (IPP). B: Leucine-proline-proline (LPP). C: Valine-proline-proline (VPP). Respective number of observations for Control, XPP, CasH and CasH +XPP are for graph A: n = 6, 9, 8 and 9; graph B: n = 6, 10, 9 and 9; graph C: n = 5, 9, 9 and 9. Values are mean ± SEM. Positive values is net release, negative values is net uptake. Statistics: repeated measures two-way ANOVA, mixed model, planned comparisons. All curves are significantly different from the XPP mixture: effect test mixture p<0.01; effect time p<0.01; interaction p<0.01

PDV fluxes showed a net release of all XPP after intra gastric administration of XPP in different matrixes (Fig 2). We observed no endogenous production of XPP by the PDV. The patterns of the curves were comparable for IPP, LPP and VPP. No differences in PDV plasma flows were observed (S2 Table. Plasma flows—Study 1.). The single dose water-based synthetic XPP matrix, showed comparable portal availability for IPP, LPP and VPP, with an average of 0.08 ± 0.01% of the intake (Table 4). The portal availability was higher in a protein (CasH) matrix than in water-based XPP matrix, but different between IPP, LPP and VPP (IPP 3.1 times, p = 0.026; LPP 1.8 times, p = 0.23; VPP 83 times, p<0.0001). For VPP, the spiked CasH showed a different portal availability in comparing to the non-spiked CasH matrix (0.05 times of CasH, p<0.0001). To understand the significant XPP effect and spike effect, data were further analyzed, by expressing the data as % of theoretically intake of XPP (S3 Table. Theoretical XPP intake and their portal availability—Study 1.), considering the tri-peptide sequences in the source of the CasH: amino acid sequence of bovine k and β casein (www.genome.jp; CASB-BOVIN, CASK-BOVIN). No spike effect of VPP was observed when corrected of potential non free available VPP sequences in the CasH. With this correction we determined that on average the portal availability was 1.8 times higher in the protein matrix (XPP 0.08 ± 0.01 vs CasH 0.14 ± 0.02, two-way ANOVA, matrix effect, p = 0.004). Further analyses of the PDV flux curves showed an increase of 2.1 times of the portal release half-life (Table 4) in the protein matrix in comparison to the water-based matrix (XPP: 15 ± 2 min vs CasH: 33 ± 3 min, two-way ANOVA, matrix effect <0.0001).

**Table 4:** Portal bioavailability and release half-life of XPP peptides—Effect of a protein matrix.

						*p-value* ^*1*^		
*Parameter*	*Matrix*	*Group*	*IPP*	*LPP*	*VPP*	*Matrix/Spike effect*	*XPP effect*	*Inter-action*
***PDV* total net balance (% of intake)**	*Water-based*	XPP	0.08±0.03	0.09±0.03	0.07±0.02	<0.0001	<0.0001	<0.0001
	*protein*	CasH	0.25±0.04*	0.17±0.03	5.81±0.98*	<0.0001	<0.0001	<0.0001
		CasH+XPP	0.19±0.03	0.16±0.03	0.28±0.05^#^			
***t***_***½*,*r***_ ***(min)***	*Water-based*	XPP	11±1	16±5	18±5	<0.0001	0.921	0.556
	*protein*	CasH	34±4^§^	32±3^§^	32±5^§^	0.297	0.678	0.739
		CasH+XPP	32±3	29±3	34±4			

Portal bioavailability, measured as post-prandial total net release to the portal system (PDV total net balance), and time when 50% of total portal tri-peptide net release occurred (t½,r) after an *intra gastric* bolus administrated of the tri-peptides isoleucine-proline-proline (IPP), leucine-proline-proline (LPP), valine-proline-proline (VPP) in a water-based matrix (synthetic XPP) or in a protein matrix (casein hydrolysate rich in XPP: CasH). Values are means ± SEM; XPP: n = 8; CasH: n = 8; CasH+XPP: n = 9. Significance: p<0.05.

^1)^: significance for comparison of water-based matrix (XPP) with protein matrix (CasH). Or significance for comparison between XPP spiked (CasH+XPP) and non-spiked protein matrix (CasH): Two-way ANOVA. When appropiate post-hoc unpaired t-test is done:

*): p<0.05 significance for comparison IPP, LPP or VPP of water-based matrix (XPP) relative to protein matrix (CasH).

^#)^: p<0.05 significance for comparison IPP, LPP or VPP of protein matrix (CasH) relative to spiked protein matrix (CasH+ XPP). When appropriate post-hoc Fisher’s LSD is done:

^§)^: p<0.05 significance for comparison IPP, LPP or VPP of waterbased matrix (XPP) relative to protein matrix (CasH).

### Multiple organ interactions of XPP peptides

**Fig 3:**
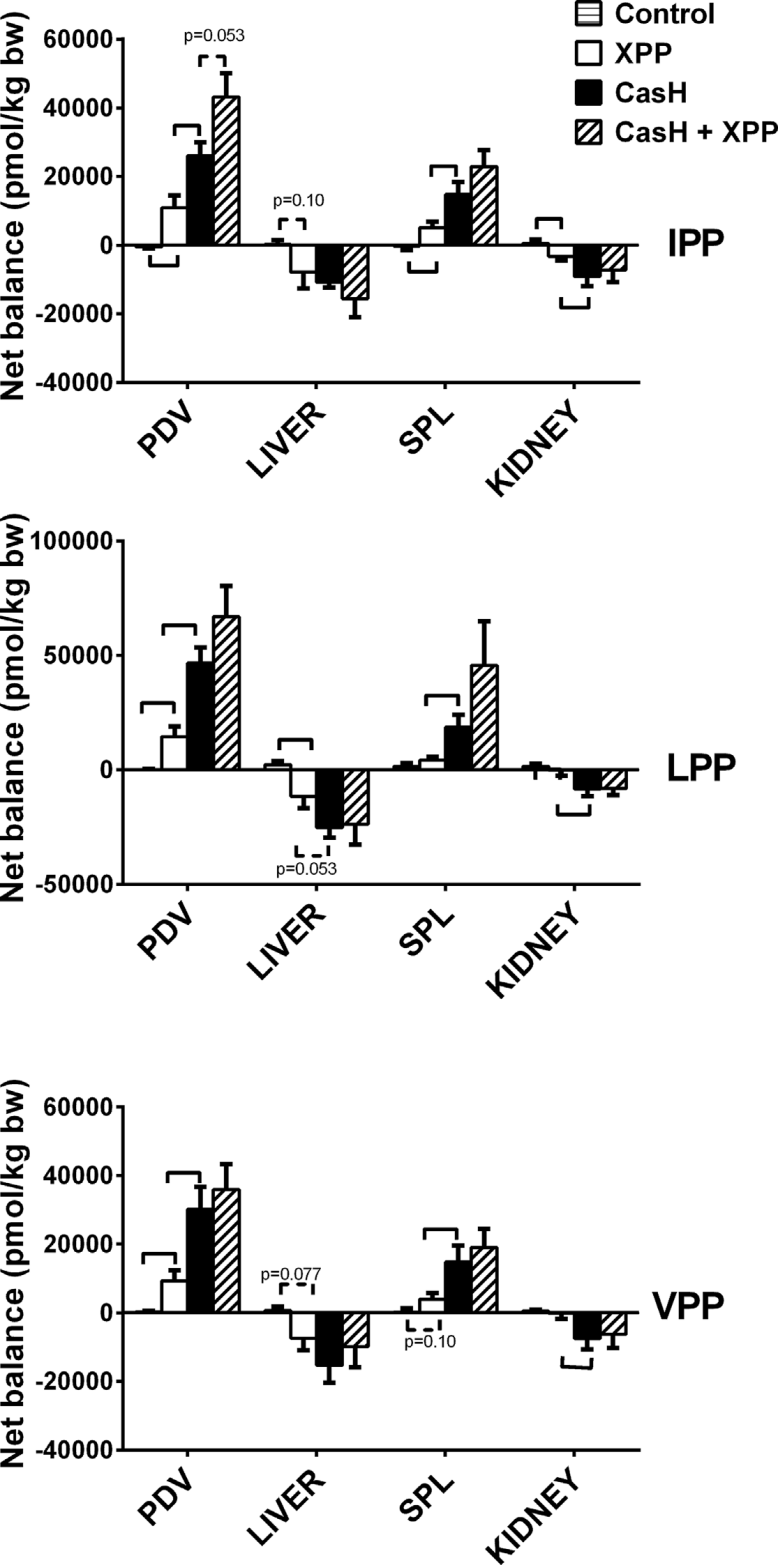
Organ Total Net balances of XPP peptides. Study 1. Post-prandial total net balances across organs over 90 min experimental period after *intra-gastric* administration of tri-peptide (XPP) mixtures: a synthetic dose of XPP (XPP), XPP containing casein hydrolyte (CasH) and spiked CasH (CasH + XPP). Organs: portal drained viscera (PDV), Liver, Splanchnic area (SPL), Kidneys. Positive balance is net release by an organ. Negative balance is net uptake by an organ. Values are mean ± SEM. Statistics: unpaired t-test for comparison of net balances of natural occurring XPP vs administrated XPP (Control vs XPP), water-based vs protein matrix (XPP vs CasH), and for comparison protein matrixes with vs without XPP spike (CasH vs CasH+XPP). Hooks: significance p<0.05. dotted hooks: tendency p<0.10.

In Study 1, we determined the XPP fluxes across multiple organs, to understand more about the influence of different organs on the systemic availability. We found no effect of the matrixes on the plasma flows of PDV, liver and kidneys (S4 Table). In the post-absorptive Control group, total net balances of XPP were not different from zero (Fig 3). Post-prandial balances showed a net release of XPP by the PDV, followed by a partial uptake by the liver, resulting in net release of XPP by the splanchnic area. Systemic available XPP are in part, taken up by the kidneys.

### Effect of meal matrix on the systemic availability of XPP peptides

In study 2, we compared XPP responses between a protein and a meal matrix and the potential effects of different macro-nutrients in a meal matrix. With the Basal meal matrix, the systemic availabilities of XPP’s were 1.2 times (p = 0.019, Wilcoxon rank test) increased over a post-prandial period of six hours in comparison to the protein matrix CasH2 (Table 5). The low quality protein and fiber were the macronutrients that increased the XPP systemic availability in a meal further with resp. 1.5 and 1.4 times in comparison to the Basal meal. (Two-way ANOVA with post-hoc Dunnett’s test; macronutrient effect: resp. p = 0.0003 and p = 0.0009).

**Table 5:** Systemic bioavailability of XPP peptides—Effect of a meal matrix.

					*Statistics*					
*Matrix*	*Group*	*IPP (%)*	*LPP (%)*	*VPP (%)*		*Matrix/diet effect*	*XPP effect*	*Interaction*		*Macronutrient effect*
***protein***	CasH2	84±11	86±8	84±14	p^1^	0.010	0.986	0.986		
***meal***	Basal	100	100	100	p^2^	0.0001	0.832	0.990		
	LQprot	160±22	152±15	143±19					p^3^	0.0003
	hCHO	105±8	109±11	100±11					p^3^	0.987
	hFat	112±14	115±16	116±18					p^3^	0.628
	Fiber	126±18	152±29	140±23					p^3^	0.009

Systemic absolute bioavailability derived from the systemic response after an intra gastric bolus administrated of the tri-peptides isoleucine-proline-proline (IPP), leucine-proline-proline (LPP), valine-proline-proline (VPP) in a protein matrix (casein hydrolysate rich in XPP: CasH2) or in CasH containing meal matrix (Basal). Or in meals with different amount of macronutrients: low quality protein (LQprot), high amount of carbohydrates (hCHO + CasH), high amount of fat (hFat) or with fiber (Fiber). Systemic bioavailability is expressed relative to Basal in %. Values are mean ± SEM. Basal, hCHO: n = 10; hFat, LQprot: n = 9; CasH2: n = 8. Significance: p<0.05.

^p^1^^: significance for comparison of protein matrix with meal matrix: Two-way ANOVA.

^p^2^^: significance for comparison meal matrixes: Two-way ANOVA.

^p^3^^: significance for macronutrient effect (LQprot, hCHO, hFat, or Fiber meal vs Basal): post-hoc Dunnett’s multiple comparison test.

### Effect of a meal matrix on portal availability of XPP peptide

Expressed as the fraction dose of total PDV net balance over the experimental period of six hours, the portal availability was increased with an average 1.8 times (56±7% of Basal, two-way ANOVA, matrix effect, <0.0001) if the XPP containing CasH was given with a complete meal (Table 6). In contrast with the systemic bioavailability, we did not observe differences between the basal and the other meals and in PDV plasma flows (S4 Table. PDV Plasma flows—Study 2.). Further analysis of the PDV flux curves showed an increase of 2.6 times of the portal release half-life (Table 7) in the meal matrix in comparison to the protein matrix (CasH2: 47 ± 5 min; Basal: 122 ± 11 min; two-way ANOVA, matrix effect <0.0001). Also, the portal release half-life was reduced in a low quality protein meal 1.5 times, and increased 1.3 times in a high fat containing meal (LQprot: 80 ± 7; hFat: 163±10; two-way ANOVA with post-hoc Dunnett’s multiple comparison, macronutrient effect resp. p = 0.009 and p = 0.014).

**Table 6:** Portal bioavailability of XPP peptides—Effect of a meal matrix.

					*Statistics*			
*Matrix*	*group*	*IPP (%)*	*LPP (%)*	*VPP (%)*		*Matrix/diet effect*	*XPP effect*	*Interaction*
***protein***	CasH2	54±12*	55±13*	59±15*	p^1^	<0.0001	0.948	0.948
***meal***	Basal	100	100	100	p^2^	0.859	0.872	0.999
	LQprot	99±9	100±12	109±10				
	hCHO	107±25	116±31	113±28				
	hFat	95±15	79±15	106±23				
	Fiber	99±29	113±54	106±32				

Portal bioavailability derived from the systemic response after an intra gastric bolus administrated of the tri-peptides isoleucine-proline-proline (IPP), leucine-proline-proline (LPP), valine-proline-proline (VPP) in a protein matrix (casein hydrolysate rich in XPP: CasH2) or in CasH containing meal matrix (Basal). Or in meals with different amount of macronutrients: low quality protein (LQprot), high amount of carbohydrates (hCHO + CasH), high amount of fat (hFat) or with fiber (Fiber). Portal bioavailability is expressed relative to Basal in %. Values are mean ± SEM. Basal: n = 10; hFat, LQprot, hCHO: n = 9; CasH2: n = 8. Significance: p<0.05.

^p^1^^: significance for comparison of protein matrix with meal matrix: Two-way ANOVA.

*): p<0.05 significance for comparison IPP, LPP or VPP portal availability of CasH2 relative to Basal with post-hoc Wilcoxon signed Rank test.

^p^2^^: significance for comparison meal matrixes: Two-way ANOVA.

**Table 7:** Portal release half-life of XPP peptides—Effect of a meal matrix.

					*Statistics*					
*Matrix*	*group*	*IPP (min)*	*LPP (min)*	*VPP (min)*		*Matrix/diet effect*	*XPP effect*	*Interaction*		*Macronutrient effect*
***protein***	CasH2	47±7*	49±9*	45±7*	p^1^	<0.0001	0.977	0.916		
***meal***	Basal	125±19	116±21	125±21	p^2^	<0.0001	0.797	0.997		
	LQprot	85±12	76±13	78±13					p^3^	0.009
	hCHO	112±16	113±21	122±19					p^3^	0.970
	hFat	166±17	168±15	155±22					p^3^	0.014
	Fiber	136±10	114±19	129±18					p^3^	0.997

Time after administration when 50% of total portal tri-peptide release (net balance) has occurred (t_½,r_ in min). Values are mean ± SEM. (Basal, LQprot, hFat: n = 10; CasH2, hCHO: n = 9; hCHO: n = 7) Significance: p<0.05.

^p^1^^: significance for comparison of comparing protein matrix with meal matrix: Two-way ANOVA.

*): p<0.05 significance for comparison IPP, LPP or VPP portal release half-life of CasH2 relative to Basal with post-hoc Sidak’s multiple comparison test.

^p^2^^: significance for comparison meal matrixes: Two-way ANOVA.

^p^3^^: significance for macronutrient effect (LQprot, hCHO, hFat, or Fiber meal vs Basal) with post-hoc Dunnett’s multiple comparison test.

## Discussion

Systemic bioavailability of orally ingested lacto-tri-peptides (XPP) may affect their potential bioactive impact. We hypothesized that macronutrients, especially protein, are able to change systemic availability and that differences in absorption kinetics of the XPP in the splanchnic region cause these changes.

Does a protein matrix improve bioavailability? In a post-absorptive state, we do not detect XPP in plasma above the limit of detection and thus any measurable XPP most likely originates from the meal (Fig 1). The calculated systemic bioavailability of XPP in the presence of a protein matrix (casein hydrolysate) does improve for all XPP, but not in the same proportion (Table 3, IPP: 4.7 times; LPP: 2.5 times; VPP: 121 times). The calculated systemic bioavailability pattern reflects the pattern of the measured portal bioavailability (Table 4), albeit underestimated because of delayed and prolonged systemic bioavailability after 90 min. This indicates that the systemic availability mainly relates to the portal availability of XPP. A protein matrix improves the portal bioavailability of all XPP by prolonging gut absorption (Fig 2, Table 4), but also not in the same proportion. The spiked protein matrix indicates that the relative large improvement of VPP bioavailability was not matrix related.

A point to consider is whether the presence of not-freely- available XPP’s (XPP’s bound in longer peptides (“encrypted”) in the casein hydrolyte affected our results. The portal bioavailability of XPP in a water-based matrix is comparable between the XPP. This in contrast with XPP in the protein matrix. We assume that this is related to encrypted VPP in the protein source of CasH. The source of CasH is κ- and β-casein and these proteins have lacto-tri-peptides encrypted [36], that are liberated during an enzymatic hydrolysis process by the manufacturer. It is likely that still encrypted XPP’s in the larger peptides of the hydrolyte are liberated during the digestive process in the pig [1]. These XPP also will contribute to the intestinal net balance. More in detail, the current enzymatic hydrolysis process (information of the manufacturer) liberates mainly VVVPP-peptides and only a small amount of VPP. Therefore, we conclude by theoretically calculations (S3 Table. Theoretical XPP intake and their portal availability—Study 1.) that the high fraction dose of VPP release in comparison to IPP and LPP relates to this mechanism. We found that the average improvement of XPP portal bioavailability by a protein matrix is 1.8 times, albeit underestimated because of prolonged portal bioavailability after 90 min.

The prolonged XPP net absorption from the protein meal could be the result of processes such as stomach emptying, digestion and competition between the absorption of XPP and amino acids on the lumen side. Some of the digested peptides in the dietary proteins are resistant to further digestion because of the type of amino acids bonds to other amino acids like for proline-containing peptides [2,37]. Trans-epithelial transport in the intestinal tract to the portal system of XPP can occur in three different ways: *para*-cellular, *trans*-cytotic or *via* peptide transport system *via* the proton-dependent transporter, PepT1 [24,38]. *In vitro* measurements of trans-epithelial flux of VPP across a Caco-2 cell monolayer [38] indicated that the *para*-cellular route is the most likely route for intact VPP. Because absorption of XPP in hydrolysates in healthy pigs is not more limited than the same amount of single amino acids [39,40], we think that delayed stomach emptying caused the prolonged absorption.

The systemic pharmacokinetics suggests that the improved bioavailability mainly is caused by delay in absorption and prolonged elimination. Systemic bioavailability is not only dependent on the portal availability but also depending on the elimination from systemic plasma to other organs. We found hepatic and renal XPP uptake (Fig 3), but uptake is approx. 75% of total PDV release. This means that other, not measured organs, also take up XPP [3]. Uptake means that XPP is available for potential ACE-inhibition targets in organs like the vascular endothelial cells or absorptive epithelial cells (kidney) [3,4], but also that XPP peptides are eliminated from the systemic by intracellular breakdown to amino acids by peptidases, or excretion to urine in the kidney. Further research in intra-organ kinetics is needed to quantify those processes.

Foltz *et al* [41] determined *in vitro* that also di-peptides with a C-terminal proline residue has ACE inhibitory effects. In a pilot analysis, we found that the plasma concentration of related di-peptides is 20–400 times higher than that of XPP (S1 Table. Dipeptide/tripeptide ratio—Study 1.). This suggest that food-derived lacto-di-peptides have a potential synergetic role in the bioactivity, but that need to be determined in another experimental design.

Does a meal improve XPP systemic availability by improved portal availability? Systemic availability increased by a meal matrix but only by 20% (Table 5) in comparison to a protein matrix. Portal availability increased 80% by a meal matrix (Table 6). Higher portal release half-life (Table 7) indicates prolonged absorption and release to the portal system by the gut in a meal matrix.

Why does a complete meal matrix improve portal XPP availability? First, it is likely that the speed of absorption of regular meals is slower than a single dose of CasH and that this prolongs absorption of peptides. Fat is known to delays gastric emptying [42]. In contrast, we did not observe any improvement of the portal bioavailability with the meals that contain a high amount of fat. Secondly, the small intestine is the interface between the gut lumen and the rest of the body and therefore controls the degree and rate of transport of amino acids coming from the meal *via* the portal vein [29]. It could be that competition of amino acids trans-epithelial transport plays a role, because the amount of amino acids from digested proteins (3.05 g/kg bodyweight, Whey and Soy isolate together with CasH) is higher in the complete meals as compared to a single dose of CasH (0.73 g/kg bodyweight). We think however that this is also unlikely as the absolute amount of protein given in the test is still relative low (30% of restricted daily intake), considering the potential absorption capacity of the gut of a pig [43]. Therefore, we need to consider stimulated gut metabolism after the meal in comparison to a protein mixture alone [19]. We found that adding carbohydrates leads to higher insulin levels, it stimulates increased protein synthesis and reduced protein breakdown, leading to more gut tissue anabolism [44,45]. Therefore, we think that reduced intracellular intestinal protein breakdown is related to the reduced XPP breakdown and thus higher transport to the portal vein. We therefore think that the gut anabolic response in the meals is the most likely mechanism to a higher portal bioavailability. Further research is needed to confirm our hypothesis.

What is the influence of the macronutrients on the systemic availability? Although total portal release of XPP is comparable during the experimental period (Table 6), systemic availability is higher with the low quality protein meal and high fiber meal (Table 5). Additionally in contrast, low protein decreases and high fat increases the portal release half-life of XPP (Table 7), suggesting that increased systemic availability can not be related to differences in intestinal XPP absorption, but suggests that other organs/metabolism are involved. For instance, we observed that soy protein leads to less anabolism [17,46]. This means that organs take up less amino acids and probably also less XPP from the systemic circulation for protein anabolism, resulting in higher systemic availability of XPP. We have no good explanation for the observation of the improved XPP systemic availability by high fiber meal. Fiber has multiple health benefits but limited info is available about a direct relation between fiber and peptide/protein metabolism [47].

### Conclusion

Quantitative measurements of trans-organ availability/kinetics provide more insight of the behavior of food-derived C-terminal lacto-tri-peptides like XPP. The present study showed that XPP in a protein matrix have a prolonged portal bioavailability. In a meal with all macronutrients present, XPP are more available albeit at a low percentage (0.2–0.3%). The digestion rate of the meal, the quality of protein, and fiber contents, mainly determine systemic XPP bioavailability after a meal.

## Supporting Information

S1 Dataset (XLSX)

S1 Table Dipeptide/tripeptide ratio—Study 1. (DOCX)

S2 Table Plasma flows—Study 1. (DOCX)

S3 Table Theoretical XPP intake and their portal availability—Study 1. (DOCX)

S4 Table PDV Plasma flows—Study 2. (DOCX)
