# Supplementary material for: Enhanced Lacto-Tri-Peptide Bio-Availability by Co-Ingestion of Macronutrients
Source: PLoS One. 2015 Jun 22;10(6):e0130638. doi: 10.1371/journal.pone.0130638 (PMC4476664; doi:10.1371/journal.pone.0130638)
Supplement: S1 Table — (DOCX) [file pone.0130638.s002.docx]

# S1 Table. Dipeptide/tripeptide ratio - Study 1.

| *Dipeptide/tripeptide* | *ratio* | | |
| --- | --- | --- | --- |
| IP/IPP | 204 | ± | 114 |
| LP/LPP | 36 | ± | 15 |
| VP/VPP | 354 | ± | 153 |
| PP/XPP | 28 | ± | 12 |
| XP/XPP | 193 | ± | 119 |

**Ratio’s between the dipeptide and tripeptide plasma concentrations.** Pilot data from 2 pigs with tripeptide concentrations above baseline in the range of 1- 30 nM. Corresponding dipeptide concentration range: 0.5 – 7 µM (n=11). Values are means ± SEM. Ratio’s were higher than 1 (Wilcoxon Rank test, p<0.01). The VP/VPP ratio was significant different from LP/LPP and PP/XPP (Mann-Whitney, p<0.05).
