# Supplementary material for: Enhanced Lacto-Tri-Peptide Bio-Availability by Co-Ingestion of Macronutrients
Source: PLoS One. 2015 Jun 22;10(6):e0130638. doi: 10.1371/journal.pone.0130638 (PMC4476664; doi:10.1371/journal.pone.0130638)
Supplement: S2 Table — (DOCX) [file pone.0130638.s003.docx]

**S2 Table. Plasma flows - Study 1.**

| *Matrix* | *Group* | *PDV* | | | *Liver* | | | | *Kidneys* | | |
| --- | --- | --- | --- | --- | --- | --- | --- | --- | --- | --- | --- |
| *water-based* | Control | 47.5 | ± | 7.5 | 71.5 | ± | 10.0 | 43.6 | | ± | 6.0 |
|  | XPP | 45.9 | ± | 4.2 | 68.6 | ± | 6.4 | 35.6 | | ± | 4.0 |
| *protein* | CasH | 54.4 | ± | 5.9 | 77.6 | ± | 8.2 | 31.9 | | ± | 4.0 |
|  | CasH + XPP | 51.7 | ± | 2.4 | 74.9 | ± | 5.8 | 33.3 | | ± | 5.5 |

**Mean plasma flows in different organs of pigs after *intra-gastric* administration of control salt solution (Control), a synthetic dose of XPP (XPP), casein hydrolysate rich in XPP (CasH) or spiked CasH (CasH + XPP).** Data are expressed as mean ± SEM in ml/kg bodyweight/min. No differences were observed between means of the XPP containing groups in each organ and Control (one-way ANOVA).
