# Supplementary material for: Enhanced Lacto-Tri-Peptide Bio-Availability by Co-Ingestion of Macronutrients
Source: PLoS One. 2015 Jun 22;10(6):e0130638. doi: 10.1371/journal.pone.0130638 (PMC4476664; doi:10.1371/journal.pone.0130638)
Supplement: S3 Table — (DOCX) [file pone.0130638.s004.docx]

**S3 Table. Theoretical XPP intake and their portal availability - Study 1.**

|  | | | |  | | | | | | | | | | | ***Statistics*** | | | | |
| --- | --- | --- | --- | --- | --- | --- | --- | --- | --- | --- | --- | --- | --- | --- | --- | --- | --- | --- | --- |
|  |  | |  |  | | |  | | | |  | | | |  | ***Matrix/*** | ***XPP*** | | ***Inter-*** |
| ***Parameter*** | ***Matrix*** | | ***Group*** | ***IPP*** | | | ***LPP*** | | | | ***VPP*** | | | |  | ***Spike*** | ***effect*** | | ***action*** |
|  |  | |  |  | | |  | | | |  | | | |  | ***effect*** |  | |  |
| ***Measured ^1)^***  ***Intake (µmol/kg)*** | *Water-based* | XPP | | 13.7 | ± | 0.4 | | 15.8 | ± | 0.5 | | 14.1 | ± | 0.3 |  |  |  |  | |
|  | *protein* | CasH | | 10.3 | ± | 0.1 | | 28.0 | ± | 0.8 | | 0.5 | ± | 0.03 |  |  |  | |  |
|  |  | CasH+XPP | | 23.1 | ± | 1.0 | | 42.6 | ± | 2.0 | | 13.3 | ± | 0.6 |  |  |  | |  |
| ***Theoretical ^2)^ Intake (µmol/kg)*** | *protein* | CasH | | 25.9 | ± | 0.3 | | 32.8 | ± | 0.9 | | 15.9 | ± | 0.8 |  |  |  |  | |
|  |  | CasH+XPP | | 38.2 | ± | 1.6 | | 67.4 | ± | 3.1 | | 21.2 | ± | 0.9 |  |  |  |  | |
| ***PDV total net balance (% of theoretical intake)*** | *Water-based* | XPP | | 0.08 | ± | 0.03 | | 0.09 | ± | 0.03 | | 0.07 | ± | 0.02 | P^1^ | 0.004 | 0.339 | 0.139 | |
|  | *protein* | CasH | | 0.10 | ± | 0.01 | | 0.14 | ± | 0.02 | | 0.19 | ± | 0.03* | P^2^ | 0.480 | 0.01 | | 0.478 |
|  |  | CasH+XPP | | 0.12 | ± | 0.02 | | 0.10 | ± | 0.03 | | 0.18 | ± | 0.04 |  |  |  | |  |

**Intake of the tri-peptides isoleucine-proline-proline (IPP), leucine-proline-proline (LPP), valine-proline-proline (VPP) in a water-based matrix (synthetic XPP) or in a protein matrix (casein hydrolysate rich in XPP: CasH).** Values are means ± SEM; XPP: n=9; CasH: n=8; CasH+XPP: n=10. ^1^) Intake is average (measured) amount of free available XPP in each test mixture.^2^) Intake is theoretically intake, considering the tri-peptide sequences in the source of the CasH: amino acid sequence of bovine k and β casein (www.genome.jp; CASB-BOVIN, CASK-BOVIN)

**Portal bioavailability measured as post-prandial total net release to the portal system (PDV total net balance) after an *intra gastric* bolus administrated of the tri-peptides isoleucine-proline-proline (IPP), leucine-proline-proline (LPP), valine-proline-proline (VPP) in a water-based matrix (XPP) or in a protein matrix (XPP containing casein hydrolysate: CasH).**Values are means ± SEM; XPP: n=8; CasH: n=8; CasH+XPP: n=9. Significance: p<0.05. Tendency: p<0.10. p^1^: significance for comparison of water-based matrix (XPP) with protein matrix (CasH): Two-way ANOVA p^2^: significance for comparison between spiked (CasH+XPP) and non-spiked protein matrix (CasH): Two-way ANOVA. When appropiate post-hoc unpaired t-test is done:*): p<0.05 significance for comparison IPP, LPP or VPP of water-based matrix (XPP) relative to protein matrix (CasH).
