# Supplementary material for: Enhanced Lacto-Tri-Peptide Bio-Availability by Co-Ingestion of Macronutrients
Source: PLoS One. 2015 Jun 22;10(6):e0130638. doi: 10.1371/journal.pone.0130638 (PMC4476664; doi:10.1371/journal.pone.0130638)
Supplement: S4 Table — (DOCX) [file pone.0130638.s005.docx]

**S4 Table. PDV Plasma flows - Study 2.**

| *Matrix* | *Group* | *PDV plasma flow* | | |
| --- | --- | --- | --- | --- |
| *protein* | CasH2 | 57.3 | ± | 8.6 |
| *meal* | Basal | 52.2 | ± | 7.2 |
|  | LQprot | 58.9 | ± | 8.9 |
|  | hCHO | 57.1 | ± | 6.6 |
|  | hFat | 58.1 | ± | 6.7 |
|  | Fiber | 50.7 | ± | 4.3 |

**Mean plasma flows of portal drained viscera (PDV) in pigs after administration of casein hydrolysate (CasH2) supplement, casein hydrolysate suppleted iso-caloric meal (Basal). Or meals with different amount of macronutrients: low quality protein (LQprot ), high amount of carbohydrates (hCHO + CasH), high amount of fat (hFat) or with fiber (Fiber).** Data are expressed as mean ± SEM in ml/kg bodyweight/min. No differences were observed between means of different matrixes: CasH2 vs Basal, unpaired t-test. Or between means of different meals (one-way ANOVA).
